# Plasma C4d levels correlate with treatment response and renal activity in proliferative lupus nephritis

**DOI:** 10.1093/rheumatology/keaf160

**Published:** 2025-04-25

**Authors:** Agneta Zickert, Caroline Grönwall, Anna Juto, Lina-Marcela Diaz-Gallo, Sepehr Sarrafzadeh Zargar, Ann Mongan, Henk-Andre Kroon, Myriam Martin, Anna M Blom, Edmund Chang, Iva Gunnarsson

**Affiliations:** Division of Rheumatology, Department of Medicine Solna, Center for Molecular Medicine, Stockholm, Sweden; Unit of Rheumatology, Karolinska University Hospital, Stockholm, Sweden; Division of Rheumatology, Department of Medicine Solna, Center for Molecular Medicine, Stockholm, Sweden; Division of Rheumatology, Department of Medicine Solna, Center for Molecular Medicine, Stockholm, Sweden; Unit of Rheumatology, Karolinska University Hospital, Stockholm, Sweden; Division of Rheumatology, Department of Medicine Solna, Center for Molecular Medicine, Stockholm, Sweden; Division of Rheumatology, Department of Medicine Solna, Center for Molecular Medicine, Stockholm, Sweden; Annexon Biosciences, Brisbane, CA, USA; Annexon Biosciences, Brisbane, CA, USA; Department of Translational Medicine, Lund University, Malmö, Sweden; Region Skåne, Laboratory Medicine, Clinical Chemistry, Malmö, Sweden; Department of Translational Medicine, Lund University, Malmö, Sweden; Region Skåne, Laboratory Medicine, Clinical Chemistry, Malmö, Sweden; Annexon Biosciences, Brisbane, CA, USA; Division of Rheumatology, Department of Medicine Solna, Center for Molecular Medicine, Stockholm, Sweden; Unit of Rheumatology, Karolinska University Hospital, Stockholm, Sweden

**Keywords:** systemic lupus erythematosus, lupus nephritis, complement, C4d

## Abstract

**Objective:**

The investigation of complement factors in lupus nephritis (LN) in relation to treatment response and the impact of underlying genetics of C4.

**Methods:**

Seventy-seven patients with active LN confirmed by a kidney biopsy and in whom second biopsies had been performed after immunosuppressive treatment were included. Complement factors C3, C4, C4d and C4d/C4 ratio were evaluated at the biopsy time points. The gene copy number variations of *C4* (*C4A* and *C4B*) were also investigated.

**Results:**

At baseline, 60 patients had class III/IV±V, proliferative LN (PLN) and 17 class V, membranous LN (MLN). Levels of C3 and C4 increased and C4d and C4d/C4 decreased after treatment (*P <* 0.0001), observed in treatment-responding PLN patients but not in MLN. C4d, C4 and C4d/C4 at second biopsies were associated with clinical response in PLN, and low C4d levels were found in PLN with histopathological response (*P =* 0.008). Renal activity index at second biopsies correlated to C4d and C4d/C4, but not to C3 or C4. *C4* gene copy number variations were not associated with clinical or histopathological response.

**Conclusion:**

All complement markers were affected by immunosuppressive therapy and associated with response to therapy. Levels of C4d and C4d/C4 at follow-up biopsies showed a strong association with both clinical and histopathological response in PLN. The correlation with elevated C4d and C4d/C4 and persisting high activity index on repeated biopsies strengthens the findings on C4d as a promising biomarker for treatment response in PLN. The C4 genetic variations did not influence C4 levels or response to treatment.

Rheumatology key messages

- Levels of C4d and C4d/C4 associate with clinical and histopathological treatment response in proliferative lupus nephritis (LN).
- Levels of C4d and C4d/C4 correlate to activity index on repeated kidney biopsies in proliferative LN.
- C4 genetic variations did not influence C4 levels or treatment response in LN.

## Introduction

Lupus nephritis (LN) is a severe manifestation of systemic lupus erythematosus (SLE), affecting up to 60% of the patients [1]. The complement system, especially the classical complement cascade, is of central importance in the pathogenesis, and complement components are commonly used to monitor disease activity [2].

Previous studies have shown that C4d, the final cleavage fragment of complement component 4 (C4) arising from complement activation, may be used as a biomarker for proliferative LN (PLN) [2, 3]. Plasma levels of C4d have been shown to discriminate LN from active non-renal SLE, and to decrease in patients responding to therapy [3]. C4d, but not C3 or C4, has been shown to forecast recurrence of LN [3]. Furthermore, C4d is expressed in kidney tissue in patients with LN [3, 4].

The production of C4 is genetically influenced by *C4* gene copy number variations, coded by the paralogous genes *C4A* and *C4B*, which encode the protein isotypes C4A and C4B. The human *C4* genes are located in the MHC class III region on chromosome 6. Since there is a strong linkage disequilibrium between *C4* gene copy numbers and certain *HLA* alleles, it has been challenging to attribute an independent risk for SLE. Nevertheless, a previous study suggested that *C4* gene copy numbers have a stronger effect than class II *HLA* alleles in the risk for SLE in European-ancestry populations [5]. The copy number of *C4* has previously been shown to correlate with C4 plasma levels, and low *C4A* gene copy numbers have been shown to associate with the presence of SSA/SSB antibodies [6].

Decreased levels of C4 are commonly seen in active LN, which is regarded as a consequence of complement consumption. To what extent low C4 production contributes to, or is a consequence of, kidney involvement is unknown.

We investigated the role of C4 activation and the *C4A/B* gene copy numbers in association with response to therapy in active LN patients in whom repeat kidney biopsies had been performed after treatment. Furthermore, we compared C3, C4 and C4d as markers of treatment response in LN subtypes.

## Methods

### Patients

The study consisted of 77 patients with active LN confirmed by a recent kidney biopsy, and in whom second biopsies were performed after induction immunosuppressive treatment. All patients were followed at the Rheumatology Department at Karolinska University Hospital and met the 1982 American College of Rheumatology (ACR) classification criteria [7], SLICC criteria [8] and the novel EULAR/ACR criteria [9] for SLE. Clinical data were collected from medical charts. Blood and urinary samples were collected on both biopsy occasions.

All patients had given their oral and written consent to participate, and the study was performed according to the Declaration of Helsinki. The study was approved by the ethical committee in Region Stockholm.

### Treatment

Treatment was given according to the treating physician’s decision. On the first biopsy occasion, 17 patients were on immunosuppressive treatment with azathioprine (AZA) (*n* = 9), methotrexate (MTX; *n* = 3), mycophenolate mofetil (MMF; *n* = 4) or cyclophosphamide (CYC; *n* = 1).

After the initial kidney biopsy, the patients were treated with CYC (*n* = 40), MMF (*n* = 22), rituximab (RTX) (*n* = 7) or AZA (*n* = 1). Four patients had combinations of CYC/MMF, two RTX/CYC and one patient was given CYC/RTX followed by MMF. All patients also received prednisolone at varying and tapering doses (Table 1).

**Table 1.** Clinical, laboratory and histopathological characteristics at first and second biopsies

Characteristic	First biopsy (*n* = 77)	Second biopsy (*n* = 76)	*P*-value
Gender, *n* (%)			
Female	66 (86)		
Male	11 (14)		
Age, median (range), years	32 (18–62)		
Ethnicity, *n*			
Caucasian	61		
Asian	7		
African	3^a^		
Hispanic	6		
Creatinine, median (range), μmol/l	80 (32–284)	74 (33–306)	0.011
Albuminuria, median (range), g/day	1.4 (0–8.4)	0.3 (0–5.3)	<0.0001
Renal histology (ISN/RPS), *n*			
Class I-II	0	18	
Class III C	0	8	
Class III-A or A/C	15	8	
Class III-A or A/C + V	8	3	
Class IV-A or A/C	29	8	
Class IV-A or A/C + V	8	5	
Class V	17	26^b^	
Activity index^c^, median (range)	5 (0–13)	1 (0–12)	<0.0001
Chronicity index^c^, median (range)	1 (0–6)	1 (0–8)	0.0014
Prednisolone at biopsy, median (range), mg/day	10 (0–60)	10 (0–70)	n.s.
Antimalarial treatment, *n* (*n* = 75)	23		
Induction treatment, *n*			
Cyclophosphamide	40		
Mycophenolate mofetil	22		
Rituximab	7		
Azathioprine	1		
Combinations of above	7		
Anti-DNA ab positive, %	85	72	
C3, median (range), g/l	0.56 (0.02–1.26)	0.786 (0.06–1.30)	<0.0001
C4, median (range), g/l	0.08 (0.003–0.44)	0.15 (0.003–0.31)	<0.0001
C4d, median (range), mg/l	0.10 (0.21–3.03)	0.59 (0.07–1.88)	<0.0001
C4d/C4, median (range)	15.06 (0.80–975,8)	4.50 (0.46–627.0)	<0.0001

a Mixed Caucasian/African.

b Three cases mixed class V+II.

c Data missing in two cases for activity index and in three cases for chronicity index. C3 and C4: complement component 3 and 4; C4d: complement component 4 degradation product; ISN/RPS: International Society of Nephrology/Renal Pathology Society classification; *n*: number of patients; n.s.: not significant.

### Evaluation of kidney function

Kidney function was determined by serum creatinine levels (µmol/l). Urine analyses included the dip-slide procedure and determination of albuminuria by 24-h urine albumin excretion or urine albumin/creatinine ratio (mg/mmol), depending on the method available at time of biopsies.

### Serology and complement measures

Anti-dsDNA antibodies were analysed by immunofluorescence using *Crithidiae luciliae* as a source of antigen or by ELISA, according to clinical routine at the time of investigation. We handled the results as dichotomous variables (positive/negative for anti-dsDNA).

Levels of C3 and C4 (g/l) were analysed in an Optilite turbidimetric analyser (Binding Site, Thermo Fisher, Birmingham, UK) with commercially available reagents for C3c and C4, performed at the Clinical Immunology Department. Undetectable levels of C4 were set to half the detection level, 0.003 g/l.

C4d levels (mg/l) were obtained using ELISA (#COMPL C4d RUO, SVAR Life Science, Malmo, SE) as previously described [3]. Additionally, we calculated the C4d/C4 ratio.

### Genotyping of C4 gene copy numbers

Genomic DNA was extracted from whole blood. C4 gene copy numbers genotyping was performed using TaqMan^®^ real-time PCR (Thermo Fisher Scientific, Waltham, MA, USA) as previously described [10]. Briefly, PCR reactions were adjusted to a final volume of 10 µl/well using 2 µl of genomic DNA (10 ng/µl), 0.5 µl of *C4A/C4B* assays (assay ID Hs07226349 cn for C4a and Hs07226350 cn for C4b from Thermo Fisher Scientific), and 0.5 µl of TaqMan Copy Number Reference Assay, human, RNase P (cat. no.: 4403326 Thermo Fisher Scientific), 2 µl of sterile water and 5 µl of TaqPath™ ProAmp™ Master Mix (cat. no.: A30865 Thermo Fisher Scientific). Real-Time PCR was performed by QuantStudio™ 7 Flex Real-Time PCR System (Thermo Fisher Scientific). Cycling conditions were 95°C for 10 min, then 40 cycles of 95°C for 15 s, followed by 60°C for 60 s. Raw data were analysed using CopyCaller™ software (Life Technologies Corporation, Foster City, CA) [11].

### Histopathological evaluation

After a first kidney biopsy and initial immunosuppressive treatment, repeated kidney biopsies were performed after a median time of 8 months (range 6–20). All biopsies were evaluated by light microscopy, immunofluorescence and electron microscopy and classified according to the International Society of Nephrology/Renal Pathology Society (ISN/RPS) classification [12], and scored for activity and chronicity indices [13].

### Evaluation of response to therapy

We evaluated both clinical and histopathological response to treatment. Clinical complete response (CCR) was defined as reaching albuminuria <0.5 g/day (by 24-h urine albumin excretion or calculated from the urine albumin/creatinine ratio) at the second biopsy, and normal or near-normal glomerular filtration rate (GFR) (within 10% of normal GFR if previously abnormal). Clinical partial response (CPR) was defined as ≥50% reduction of albuminuria to sub-nephrotic levels and normal or near-normal GFR [14, 15].

The second biopsy was used to evaluate the histopathological response to treatment. We defined ISN/RPS class I, II or IIIC as histopathological complete response (HCR). Reduction ≥50% of activity index to ≤3 was defined as histopathological partial response (HPR). Patients not reaching the definitions for either CHR or PHR were regarded as histopathological non-responders (HNR).

### Statistics

Continuous variables are described as median and range. Categorical variables are presented as numbers and percentages. Wilcoxon’s pair test was used for comparisons of variables at baseline and follow-up. The Mann–Whitney test was used for comparisons of variables between two groups. Correlations were calculated using Spearman’s rank correlation. Area under the curve (AUC) including 95% CI of receiver operating characteristics (ROC) curves were determined to evaluate the performance of the different complement components and C4d/C4 ratio on the classification of responders and non-responders. *P*-values <0.05 were considered statistically significant. Analyses were performed in the Statistica software program (Statsoft Scandinavia, Uppsala, SE) and IBM SPSS Statistics for Windows, version 28.0 (IBM Corp., Armonk, NY, USA).

## Results

### Evaluation of renal disease activity and complement

At first biopsies, median creatinine was 80 µmol/l (32–284) which decreased to 74 µmol/l (33–306) at second biopsies (*P <* 0.01). The albuminuria decreased from a median of 1.4 g/day (0–8.4) to 0.3 g/day (0–5.3) at second biopsies (*P <* 0.0001).

At first biopsies, median C3 levels were 0.56 g/l (0.02–1.26), which increased at follow-up to 0.79 g/l (0.06–1.33) (*P <* 0.0001), and C4 levels were 0.07 g/l (0.003–0.44) which increased to 0.15 g/l (0.003–0.31) (*P <* 0.0001). Levels of C4d at baseline were 0.97 mg/l (0.21–3.03) with a decrease at follow-up to 0.59 mg/l (0.07–1.88) (*P <* 0.0001), and C4d/C4 ratio decreased from 15.06 (0.80–976) to 4.50 (0.46–497) (*P <* 0.0001) (Table 1).

There was no difference in levels of C3, C4 or C4d in relation to the immunosuppressive treatment given (data not shown).

### Copy number variation results

**Figure 1.**
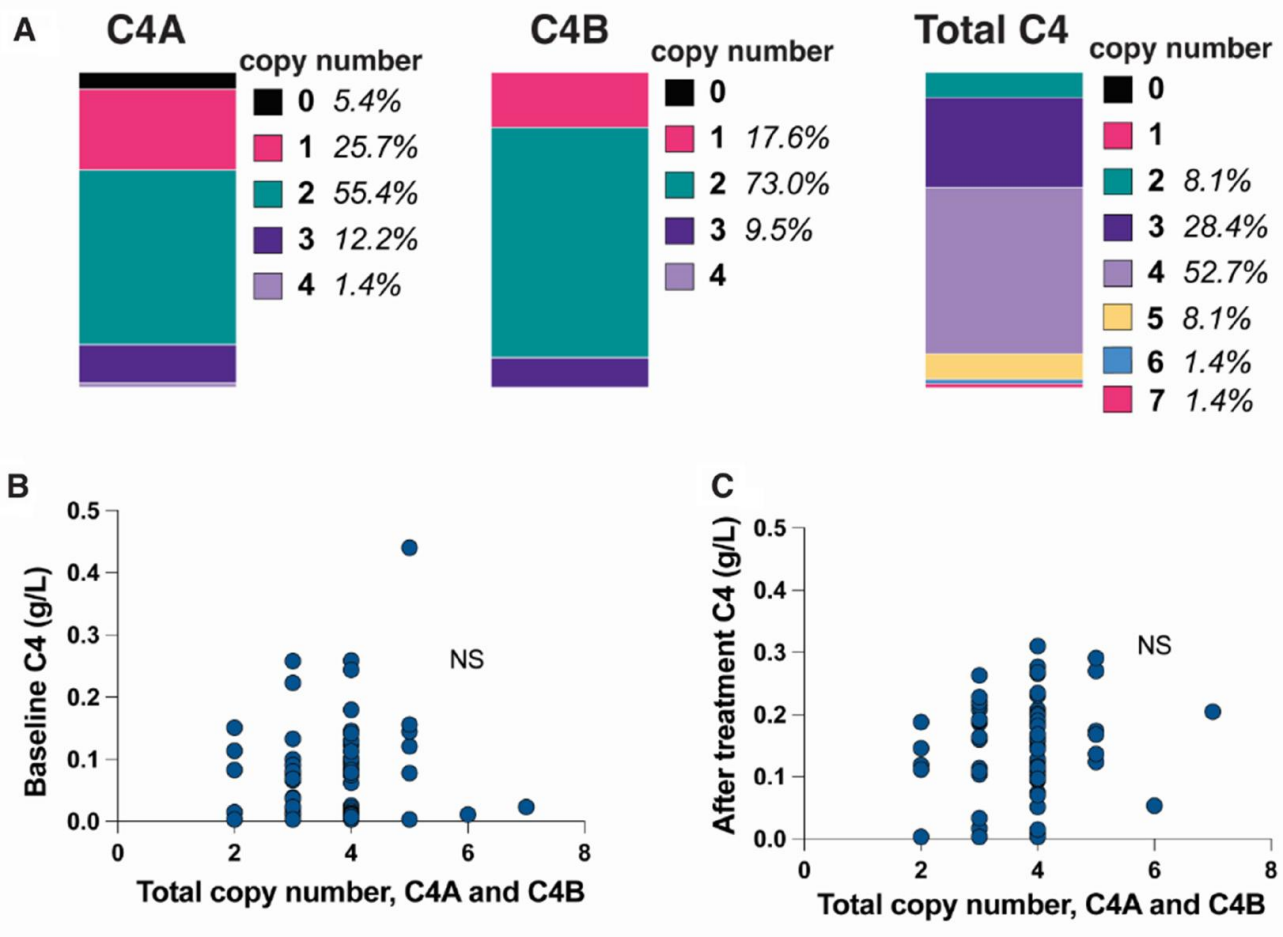
*C4* copy number variation in relation to C4 levels at baseline and repeat biopsy. (**A**) Distribution of copy numbers of *C4A*, *C4B* and total number of *C4* copies. (**B**) Total *C4* copy numbers in relation to C4 levels in plasma at baseline evaluation. (**C**) Total *C4* copy numbers in relation to C4 levels in plasma at the repeat biopsy time point. n.s.: not significant

Data on gene copy numbers (GCN) of *C4A* and *C4B* were available in 74 patients. Forty-one patients had two *C4A* GCN (55.4%) and 54 carried two copies of *C4B* (72.9%). All patients had at least one *C4* gene copy. Combining *C4A* and *C4B* GCN, six patients had two copies, 21 had three, 39 had four, six had five, and one patient had six and seven copies each. There was no difference in *C4* GCN and gender. In patients with a first renal flare (*n* = 54) and available information on GCN (*n* = 52), there was no difference in *C4A* GCN and age at first renal flare (data not shown). Results for number of *C4A* and *C4B* copies and total number are presented in Fig. 1A.

There was no association between the total number of *C4* copies (*C4A* and *C4B* combined) and C4 levels at the two biopsy occasions (n.s., Fig. 1B and C). No association was found between the number of *C4A* and *C4B* GCN and C4d or the C4d/C4 ratios (data not shown).

### Histopathological evaluation

At first biopsies, 60 patients had PLN (class III/IV±V) and 17 had membranous nephritis (MLN) (class V).

In all patients, the activity index decreased after immunosuppression (*P <* 0.0001). Despite treatment, there was an increase in the chronicity index (*P =* 0.0014) from baseline to second biopsies (Table 1).

### Evaluation of clinical and histopathological response to therapy

#### Clinical response

When evaluating the clinical response, 38 patients achieved CCR, 16 CPR and 22 were clinical non-responders (CNR). Among patients with PLN, 37 were CCR, 11 CPR and 11 CNR. In MLN, there was one CCR, five CPR and 11 CNR. For statistical reasons we divided the response groups into two: clinical responders (CR) (CCR and CPR combined) *vs* non-responders (NR).

### Clinical response in relation to C3, C4, C4d and C4d/C4

In the total patient population, there was no difference in levels of C3, C4, C4d or C4d/C4 at first biopsies in relation to clinical response. At second biopsies, C4 and C3 levels were higher in patients achieving CR compared with NR (*P =* 0.010 and 0.013, respectively) and C4d/C4 was lower in CR *vs* NR (*P =* 0.016) but no differences were seen in C4d levels between the clinical response groups.

Next, we evaluated patients with PLN and MLN separately. In PLN, C4 levels were higher (*P =* 0.010) whereas both C4d levels and C4d/C4 were lower at second biopsies in CR *vs* NR (*P =* 0.046 and 0.006, respectively), but no difference was found for C3 (n.s.). In MLN, there were no differences in levels of C3, C4, C4d or C4d/C4 in relation to clinical response (Supplementary Table 1, available at *Rheumatology* online).

**Figure 2.**
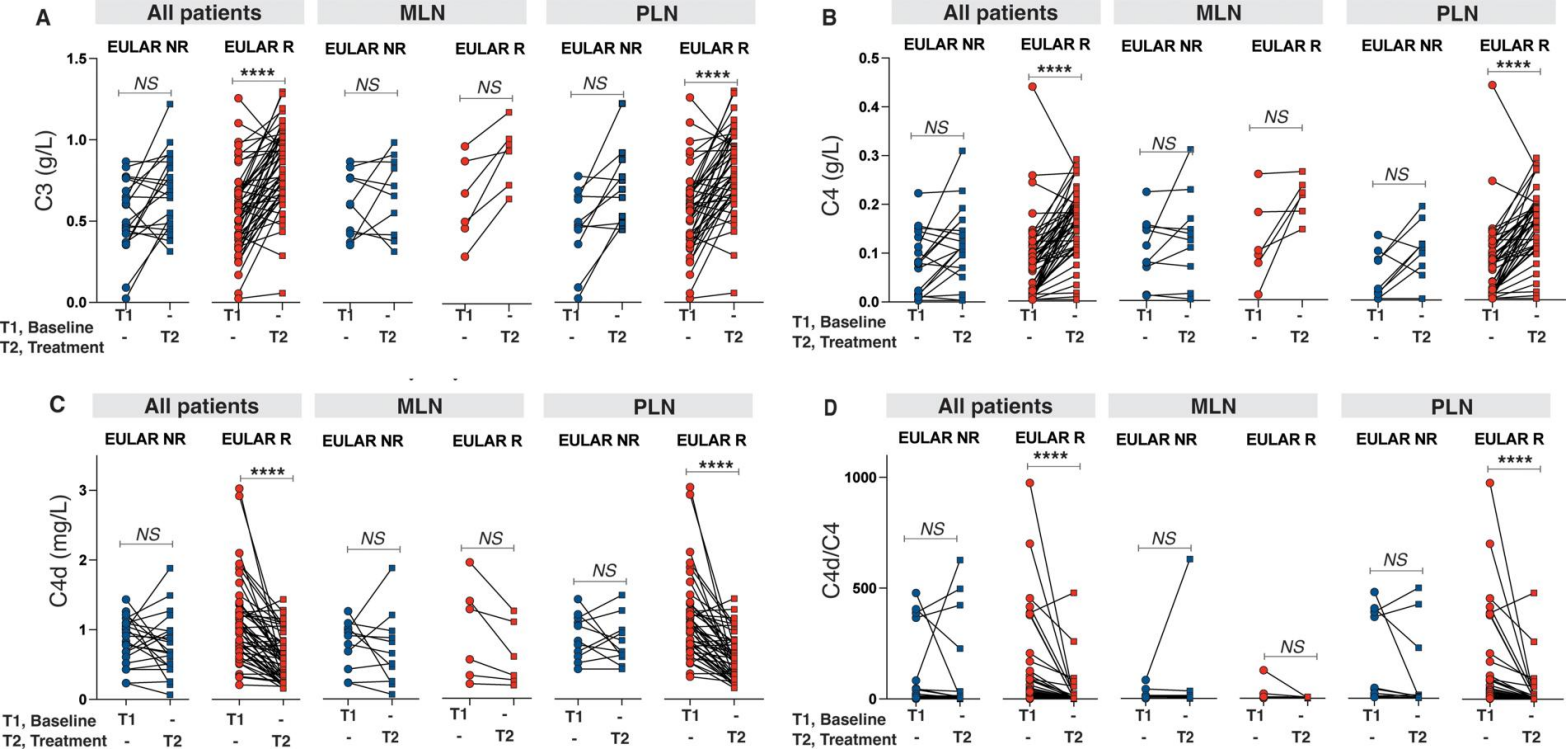
Complement levels at baseline and repeat biopsies in relation to clinical treatment response in different patient subgroups using the EULAR response criteria [14]. (**A**) C3 levels at baseline and repeat biopsy in all patients, MLN and PLN. (**B**) C4 levels at baseline and repeat biopsy in all patients, MLN and PLN. (**C**) C4d levels at baseline and repeat biopsy in all patients, MLN and PLN. (**D**) C4d/C4 ratio at baseline and repeat biopsy in all patients, MLN and PLN. ****P <0.0001. MLN: membranous lupus nephritis (ISN/RPS class V); NR: non-responders; n.s.: non-significant; PLN: proliferative lupus nephritis (ISN/RPS class III/IV±V); R: responders

Comparing complement levels between first and second biopsies in all patients, an increase in C3 and C4, and a decrease in C4d and C4d/C4 were found (Table 1). However, subdividing the patients in relation to clinical response, the increase in C3 and C4 and decrease in C4d and C4d/C4 were found in responders with PLN only, and no changes in complement levels were associated with response in MLN (Fig. 2).

Performing AUC and ROC curve analysis for complement levels at second biopsies in all patients in relation to clinical response showed relatively modest but significant results in terms of AUC values. Except for C4d, levels of C3 [AUC 0.686 (95% CI: 0.554, 0.818), *P =* 0.013] and C4 [AUC 0.695 (95% CI: 0.558, 0.831), *P =* 0.009] and C4d/C4 [AUC 0.682 (95% CI: 0.531, 0.833), *P =* 0.015] all indicated clinical response (Supplementary Fig. 1A, available at *Rheumatology* online).

Within the PLN patient subpopulation, however, the AUC value was improved for levels of C4 [AUC 0.750 (95% CI: 0.597, 0.904), *P =* 0.01] and C4d [AUC 0.695 (95% CI: 0.546, 0.844), *P =* 0.045], and was most strongly associated for C4d/C4 [AUC 0.770 (95% CI: 0.625, 0.914), *P =* 0.006]. No significant results were seen for C3 (Supplementary Fig. 1B, available at *Rheumatology* online).

No significant correlations were seen in MLN. Using complement levels at the first biopsy time point, no significant findings were observed in either PLN or MLN (data not shown).

### *C4* gene copy numbers and clinical response

There were no significant associations between GCN of *C4A* or *C4B* or *C4A* and *C4B* combined with clinical responses (Supplementary Fig. 2, available at *Rheumatology* online).

#### Histopathological response

The histopathological response was evaluated in 76 kidney biopsies. Twenty-six patients had HCR, 32 had HPR and 18 HNR. Among PLN patients, 25 were HCR, 23 HPR and 12 HNR. In MLN, there was one HCR, nine HPR and six HNR (data missing in one). For statistical reasons we divided the response groups into two groups: histopathological responders (HR) (HCR and HPR combined) *vs* non-responders (HNR).

### Histopathological response in relation to C3, C4, C4d and C4d/C4

In all study patients, there was no significant difference in C3, C4 and C4d levels or C4d/C4 at either baseline or follow-up biopsies between the histopathological response groups. However, PLN patients reaching HR had significantly lower levels of C4d *vs* NHR (*P =* 0.008) at follow-up biopsies. No differences in complement levels were seen in relation to histopathological response in MLN (Supplementary Table 2, available at *Rheumatology* online).

**Figure 3.**
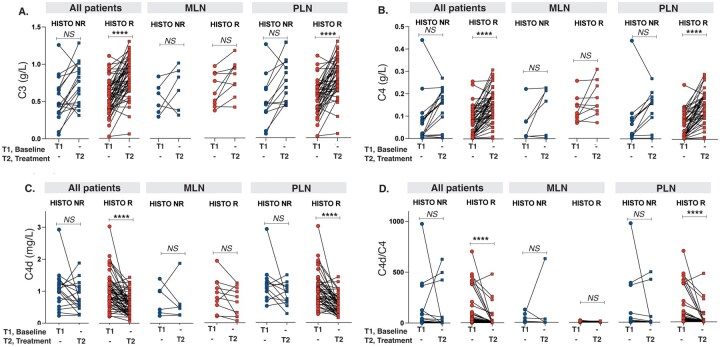
Complement levels at baseline and repeat biopsies in relation to histopathological treatment response in different patient subgroups. (**A**) C3 levels at baseline and repeat biopsy in all patients, MLN and PLN. (**B**) C4 levels at baseline and repeat biopsy in all patients, MLN and PLN. (**C**) C4d levels at baseline and repeat biopsy in all patients, MLN and PLN. (**D**) C4d/C4 ratio at baseline and repeat biopsy in all patients, MLN and PLN. ****P <0.0001. MLN: membranous lupus nephritis (ISN/RPS class V); NR: non-responders; n.s.: non-significant; PLN: proliferative lupus nephritis (ISN/RPS class III/IV±V); R: responders

Comparing complement levels at first and second biopsies, an increase in C3 and C4, and a decrease in C4d and C4d/C4 was found as stated above (Table 1). However, subdividing by PLN and MLN, and by histological response, the increase in C3 and C4 and decrease in C4d and C4d/C4 were only seen in PLN achieving HR (Fig. 3).

**Figure 4.**
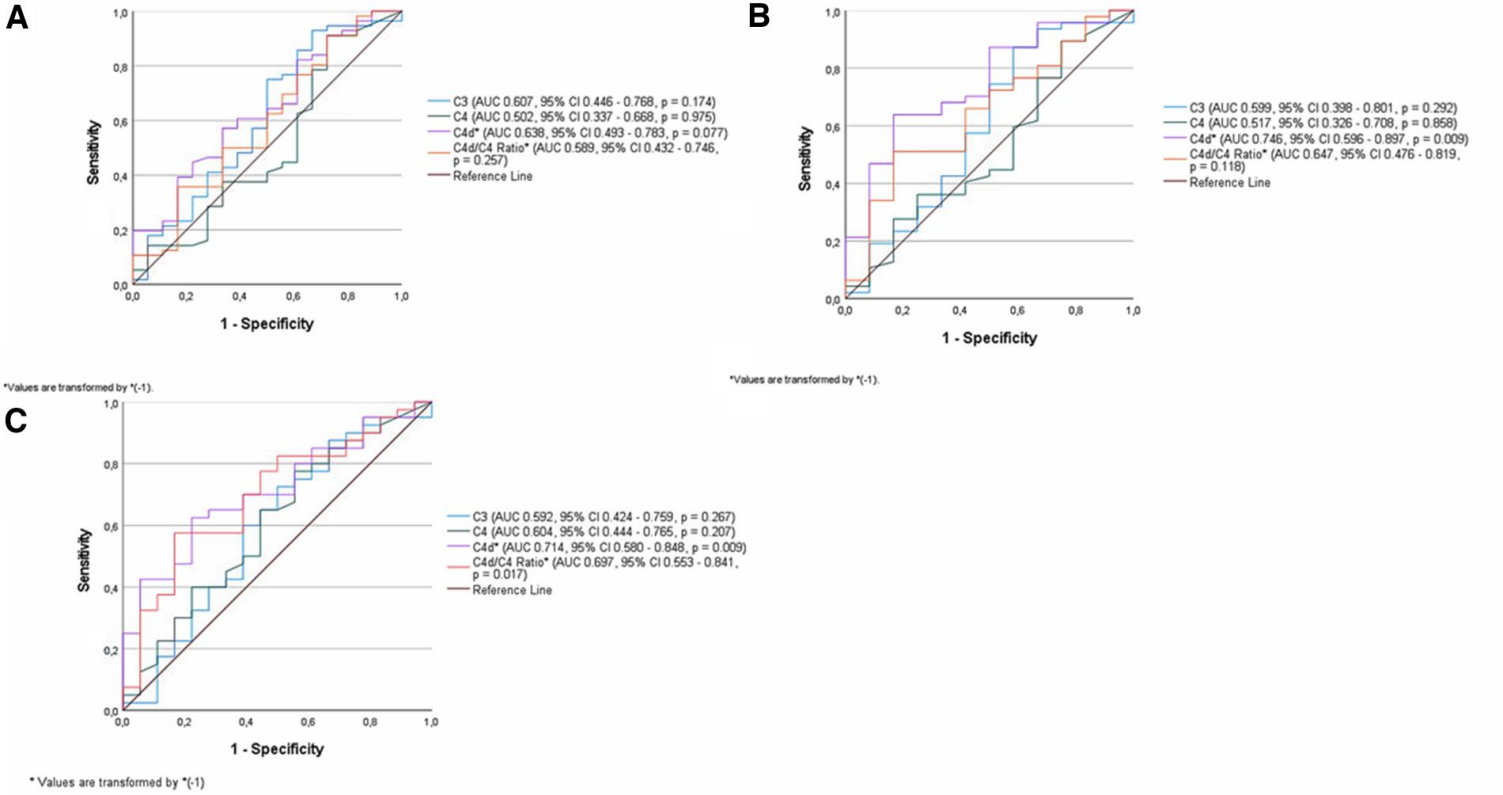
(**A**) AUC, ROC and CI for complement variables in all patients in relation to achieving a histopathological treatment response using complement levels at second biopsy. C3: AUC 0.607 (95% CI: 0.446, 0.768), *P =* 0.174; C4 AUC 0.502 (95% CI: 0.337, 0.668), *P =* 0.975; C4d: AUC 0.638 (95% CI: 0.493, 0.783), *P* = 0.077; C4d/C4: AUC 0.589 (95% CI: 0.432, 0.746), *P =* 0.257. (**B**) AUC, ROC and CI for complement variables in patients with PLN in relation to achieving a histopathological treatment response using complement levels at second biopsy. C3: AUC 0.599 (95% CI: 0.398, 0.801), *P =* 0.292; C4: AUC 0.517 (95% CI: 0.326, 0.708), *P =* 0.858; C4d: AUC 0.750 (95% CI: 0.601, 0.899), *P* = 0.008; C4d/C4: AUC 0.647 (95% CI: 0.476, 0.819), *P =* 0.118. (**C**) AUC, ROC and CI for complement variables at second biopsies in patients with PLN achieving both a clinical and histopathological response. C3: AUC 0.592 (95% CI: 0.424, 0.759), *P =* 0.27; C4: AUC 0.604 (95% CI: 0.444, 0.765), *P* = 0.21; C4d: AUC 0.714 (95% CI: 0.580, 0.848), *P* = 0.009; C4d/C4 ratio: AUC 0.697 (95% CI: 0.553, 0.841), *P* = 0.017. AUC: area under the curve; PLN: proliferative lupus nephritis; ROC: receiver operating characteristic

AUC and ROC curve analysis of complement levels at second biopsies in the total patient group achieving HR revealed no statistical significance for changes in levels of C3, C4 or C4d/C4 but a trend was seen for C4d (AUC 0.638, *P =* 0.077) (Fig. 4A). However, a significant association for C4d [AUC 0.750 (95% CI: 0.601, 0.899), *P =* 0.008] was noted in PLN patients reaching histopathological response. No significant findings were seen for C3, C4 or the C4d/C4 ratio (Fig. 4A and B). No significant findings were observed for complement variables at first biopsies.

### Anti-DNA and complement levels in relation to histopathological response

In PLN patients with positive anti-DNA (any method) at second biopsies, high levels of C4d and C4d/C4 were found in patients with HNR (*P =* 0.007 and 0.04, respectively) *vs* HR, which was not found for C3 or C4 and not among MLN patients (n.s.).

### Combined clinical and histopathological response in relation to C3, C4, C4d and C4d/C4

Comparing PLN patients achieving both clinical and histopathological responses with non-responders or patients achieving either clinical or histopathological response only, significant changes were found for C4d [AUC 0.714 (95% CI: 0.580, 0.848), *P =* 0.009] and C4d/C4 ratios [AUC 0.697 (95% CI: 0.553, 0.8481), *P =* 0.017] while not observed for C3 and C4 levels (n.s.) (Fig. 4C).

### Activity and chronicity index

No correlations between C3, C4, C4d or C4d/C4 and activity index were found at first biopsies. At second biopsy, both C4d and C4d/C4 correlated to renal activity index (*r* = 0.34 and 0.31, respectively, *P <* 0.05). A weak negative correlation was also found for C3 and activity index (*r* = −0.25, *P <* 0.05) but no correlation was found for C4 (n.s.).

**Figure 5.**
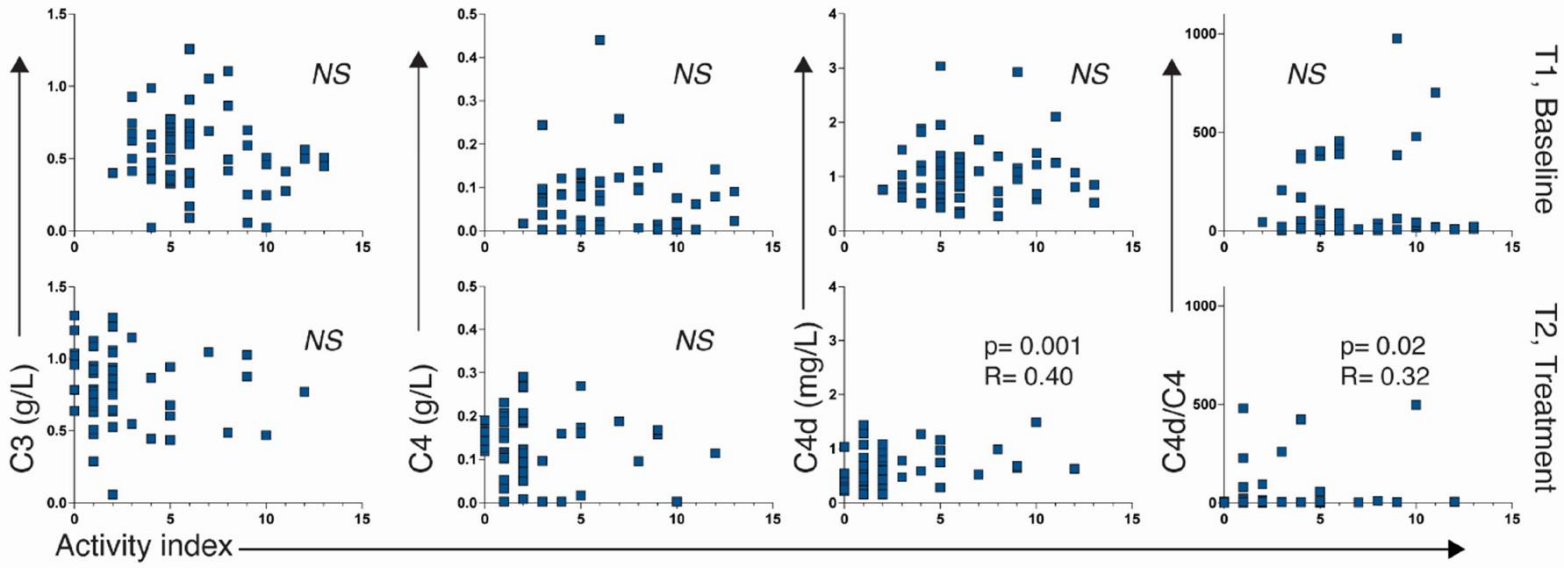
Complement levels C3, C4, C4d and C4d/C4 ratio in correlation to renal activity index [13] at T1 (first kidney biopsies) and T2 (second kidney biopsies) in patients with proliferative lupus nephritis

When subdividing PLN *vs* MLN, C4d and C4d/C4 correlated to activity index (*r* = 0.40, *P =* 0.001 and *r* = 0.32, *P =* 0.02, respectively) at second biopsies in PLN. No correlation was seen for C3 or C4. In MLN, no correlations were found either at first or second biopsies (n.s.) (Fig. 5).

In PLN, patients with the highest C4d levels (25th upper percentile) had a higher activity index at second biopsies as compared with those with the 25th percentile lowest levels (*P =* 0.01).

### *C4* gene copy numbers and histopathological response

There was no significant difference in histopathological response regarding *C4* GCN of either *C4A* or *C4B* or the total number of *C4* copies (n.s.) (Supplementary Fig. 2, available at *Rheumatology* online).

## Discussion

We found increasing levels of C3 and C4 and decreasing levels of C4d and C4d/C4 after immunosuppressive treatment in PLN patients associated with favourable clinical and histopathological responses. Low C4d levels at the time of follow-up biopsies were most strongly associated with histopathological response in PLN, and correlated with low activity index in renal tissue. No single complement component at baseline was predictive of either clinical or histopathological response. The C4 genetics was not found to influence C4 levels or outcome.

Levels of C3 and C4 are the net result of complement consumption and rate of synthesis, whereas C4d is exclusively generated upon C4 activation, which strengthens its suitability as a specific marker of complement activation. Here, changes in all investigated complement markers were shown to associate with both clinical and histopathological response to treatment following immunosuppression. The association was however limited to PLN when the LN subtypes were analysed separately.

Using levels of complement at the second biopsy as markers of clinical response in the entire study population, we found associations for C3, C4 and C4d/C4, but not for C4d. Restricting to PLN only, however, an association with clinical response was seen primarily for C4d levels, and to a lesser extent C4 and C4d/C4, while C3 levels failed to reach statistical significance.

A similar pattern was seen for histopathological response. Here, no association was seen in the entire patient group, but when subdividing by LN subtypes, a strong association between low C4d and response was seen in PLN. These findings, combined with the correlation between renal activity index and C4d and C4d/C4 at second biopsies, indicate that measurement of C4d can be a promising new tool for evaluation of treatment response in PLN. However, an appropriate cut-off value needs to be defined in larger study cohorts. None of the complement variables at baseline could predict either clinical or histopathological response.

The gene encoding C4 is polymorphic and consists of two coding gene variants, *C4A* and *C4B*, which have been shown to have different roles. C4A protein has been shown to increase the efficiency in clearance of immune complexes while C4B protein was found to be more efficient in the targeting of microbes [16]. Low *C4A* gene copy numbers have been reported to be a strong risk factor for systemic autoimmune diseases including SLE whereas the effect of *C4B* is smaller [6]. The median number of *C4A* copies in the general population is two [5], a finding also observed in this LN population with 55% of patients carrying two copies. However, in this study *C4A* copy numbers were not associated with levels of C4 protein either at baseline or repeat biopsy and, more importantly, not associated with clinical or histopathological response to treatment. Thus, subnormal *C4* GCN do not seem to influence the effects of immunosuppressive therapy in a LN population.

In a previous publication, there was a strong association between *C4A* copy number and the presence of autoantibodies against SSA/Ro and SSB/La in systemic inflammatory autoimmune diseases, including SLE [6]. The total number of *C4* copies also correlated with C4 levels in plasma. The divergent findings could be explained by differences in sample size and study populations, where we included patients with active disease and exclusively patients with kidney involvement.

In another study, a low (one) *C4* copy number, when associated with a heterogeneous C2 deficiency, was shown to increase the risk for the development of SLE and primary Sjögren’s syndrome. Furthermore, a lower age at diagnosis was noted in patients with this genetic combination, including a trend toward earlier onset of nephritis compared with patients with normal C2 levels [17]. We could not verify that low GCN was associated with age at nephritis onset (data not shown), and no data on C2 deficiency were available for this cohort.

The lack of association of *C4* copy numbers and LN presented here, combined with previous reports on association with SSA/SSB, suggests that C4 genetics may be a contributing pathogenetic factor in other, mainly non-renal SLE subtypes. In a recent study, SSA/SSB positivity was associated with a Sjögren’s disease like SLE subgroup with HLA-DRB1*3. In contrast, LN was mainly present in individuals positive for anti-nucleosome/SmRNP/DNA/RNPA autoantibodies and associated with HLA-DRB1*15 [18].

This is a retrospective study in a clinical setting, associated with limitations that may impede the results. Immunosuppressive therapy was given according to the physicians’ decisions, and treatment recommendations have changed over time, which may have affected both clinical and histopathological outcome. Different assays for determination of anti-DNA antibody detection were used, which limited the possibility to fully investigate associations with anti-DNA in this study. Second renal biopsies were performed as a clinical routine where the time point was decided by the treating physician and not based on predefined criteria. Regardless of these limitations, our results still point to C4d as a superior marker of treatment response in patients with PLN compared with the conventionally used complement factors.

Previous studies have shown a discrepancy between clinical *vs* histological response to treatment, which suggests that patients may have ongoing inflammatory activity in renal tissue despite clinical quiescent disease [19]. A major strength of this study is having access to repeat biopsies, which provide a unique opportunity to study the role of complement in association to renal histopathological activity and thereby evaluate treatment response also at a tissue level. Evaluations of clinical and histopathological response are based on different methods and may reflect different aspects of lupus kidney activity. Using a combination of clinical and histopathological response in comparison with the complement variables studied, C4d and C4d/C4 clearly outperformed C3 and C4 in predicting response.

We have previously shown that C4d/C4 was a promising marker for active lupus nephritis [3]. In the current study, using more patient material, also including the underlying C4 genetics, we could further demonstrate the role of C4d in different types of LN, proliferative *vs* membranous. Our findings from this extended study show that both C4d and C4d/C4 are better biomarkers than the currently used C3 and C4 for evaluation of the inflammatory state in LN, also at a tissue level. Yet, the lack of association between *C4* copy number and nephritis severity or response to treatment indicates that complement consumption rather than production better reflects nephritis pathology and is more important for treatment outcome.

In summary, measurements of C4d and C4d/C4 may be used as novel biomarkers for treatment response in LN, especially in PLN. We did not confirm *C4* genetics to contribute to C4 levels, or to be involved in achieving response to treatment in LN.

## Supplementary Material

keaf160_Supplementary_Data

## Data Availability

Data are available upon reasonable request to the corresponding author but will not be shared publicly. For all types of access, a research proposal must be submitted for evaluation by the responsible researchers of the study sites.
