# Supplementary material for: Plasma C4d levels correlate with treatment response and renal activity in proliferative lupus nephritis
Source: Rheumatology (Oxford). 2025 Apr 25;64(8):4825–33. doi: 10.1093/rheumatology/keaf160 (PMC12316364; doi:10.1093/rheumatology/keaf160)

**Supplementary Table S1. Levels of C3, C4, C4d and C4d/C4 at first and second biopsies in relation to clinical response**

|  | CR/PR | NR | p-value |
| --- | --- | --- | --- |
| C3, first biopsy  PLN  MLN | 0.57 (0.02-1.26)  0.57 (0.02-1.26)  0.59 (0.29-0.97) | 0.48 (0.02-0.87)  0.47 (0.02-0.77)  0.60 (0.11-0.87) | ns  ns  ns |
| C3, second biopsy  PLN  MLN | 0.87 (0.06-1.30)  0.82 (0.06-1.30)  0.96 (0.64-1.18) | 0.69 (0.31-1.22)  0.69 (0.45-1.22)  0.69 (0.31-0.98) | *0.013*  ns  ns |
| C4, first biopsy  PLN  MLN | 0.08 (0.003-0.44)  0.07 (0.003-0.44)  0.10 (0.01-0.26) | 0.07 (0.003-0.22)  0.02 (0.003-0.13)  0.08 (0.003-0.22) | ns  ns  ns |
| C4, second biopsy  PLN  MLN | 0.16 (0.003-0.29)  0.16 (0.003-0.29)  0.22 (0.14-0.26) | 0.11 (0.003-0.31)  0.10 (0.003-0.19)  0.13 (0.003-0.31) | *0.010*  *0.010*  ns |
| C4d, first biopsy  PLN  MLN | 1.03 (0.21-3.03)  1.03 (0.32-3.03)  0.92 (0.21-1.95) | 0.87 (0.23-1.43)  0.83 (0.43-1.43)  0.91 (0.23-1.26) | ns  ns  ns |
| C4d, second biopsy  PLN  MLN | 0.57 (0.16-1.44)  0.57 (0.16-1.44)  0.46 (0.19-1.26) | 0.68 (0.07-1.88)  0.68 (0.43-1.49)  0.66 (0.07-1.88) | ns  *0.046*  ns |
| C4d/C4, first biopsy  PLN  MLN | 15.5 (0.80-975.8)  18.1 (2.21-975.8)  6.32 (0.80-127.0) | 13.4 (1.04-478.1)  42.5 (5.31-478.1)  11.2 (1.04-263) | ns  ns  ns |
| C4d/C4, second biopsy  PLN  MLN | 3.60 (0.60-479.7)  3.69 (0.60-479.7)  2.5 (0.71-6.04) | 8.26 (0.46-627.0)  8.65 (2.24-497.1)  6.5 (0.46-627.0) | *0.016*  *0.006*  ns |

C3 and C4= Complement component 3 and 4; C4d= Complement component 4 degradation product; PLN=proliferative lupus nephritis; MLN= membranous lupus nephritis

**Supplementary Table S2. Levels of C3, C4, C4d and C4d/C4 at first and second biopsies in relation to histopathological response**

|  | HR | HNR | p-value |
| --- | --- | --- | --- |
| C3, first biopsy  PLN  MLN | 0.57 (0.02-1.10)  0.57 (0.02-1.10)  0.55 (0.11-0.97) | 0.45 (0.06-1.26)  0.45 (0.06-1.26)  0.53 (0.29-0.83) | ns  ns  ns |
| C3, second biopsy  PLN  MLN | 0.80 (0.06-1.30)  0.79 (0.06-1.30)  0.86 (0.41-1.18) | 0.72 (0.31-1.28)  0.72 (0.44-1.28)  0.74 (0.31-1.01) | ns  ns  ns |
| C4, first biopsy  PLN  MLN | 0.08 (0.003-0.26)  0.05 (0.003-0.26)  0.11 (0.003-1.18) | 0.04 (0.003-0.44)  0.04 (0.003-0.44)  0.04 (0.10-0.22) | ns  ns  ns |
| C4, second biopsy  PLN  MLN | 0.14 (0.003-0.31)  0.05 (0.003-0.29)  0.11 (0.003-0.31) | 0.16 (0.003-0.27)  0.16 (0.003-0.27)  0.19 (0.003-0.23) | ns  ns  ns |
| C4d, first biopsy  PLN  MLN | 0.90 (0.21-3.03)  0.90 (0.27-3.03)  0.87 (0.21-1.95) | 1.04 (0.23-2.93)  1.13 (0.52-2.93)  0.72 (0.23-1.40) | ns  ns  ns |
| C4d, second biopsy  PLN  MLN | 0.57 (0.07-1.44)  0.55 (0.16-1.44)  0.74 (0.07-1.26) | 0.66 (0.26-1.88)  0.86 (0.28-1.50)  0.49 (0.26-1.88) | ns  *0.008*  ns |
| C4d/C4, first biopsy  PLN  MLN | 14.43 (0.80-701.3)  21.3 (3.23-701.3)  8.52 (0.80-269.0) | 22.63 (1.04-975.8)  22.63 (2.21-975.8)  28.05 (1.04-127.0) | ns  ns  ns |
| C4d/C4 second biopsy  PLN  MLN | 4.24 (0.46-479.7)  3.98 (0.60-479.7)  5.35 (0.46-14.0) | 4.76 (1.04-627.0)  5.38 (1.04-497.2)  2.74 (1.12-627) | ns  ns  ns |

C3 and C4= Complement component 3 and 4; C4d= Complement component 4 degradation product; PLN=proliferative nephritis; MLN= membranous nephritis

**
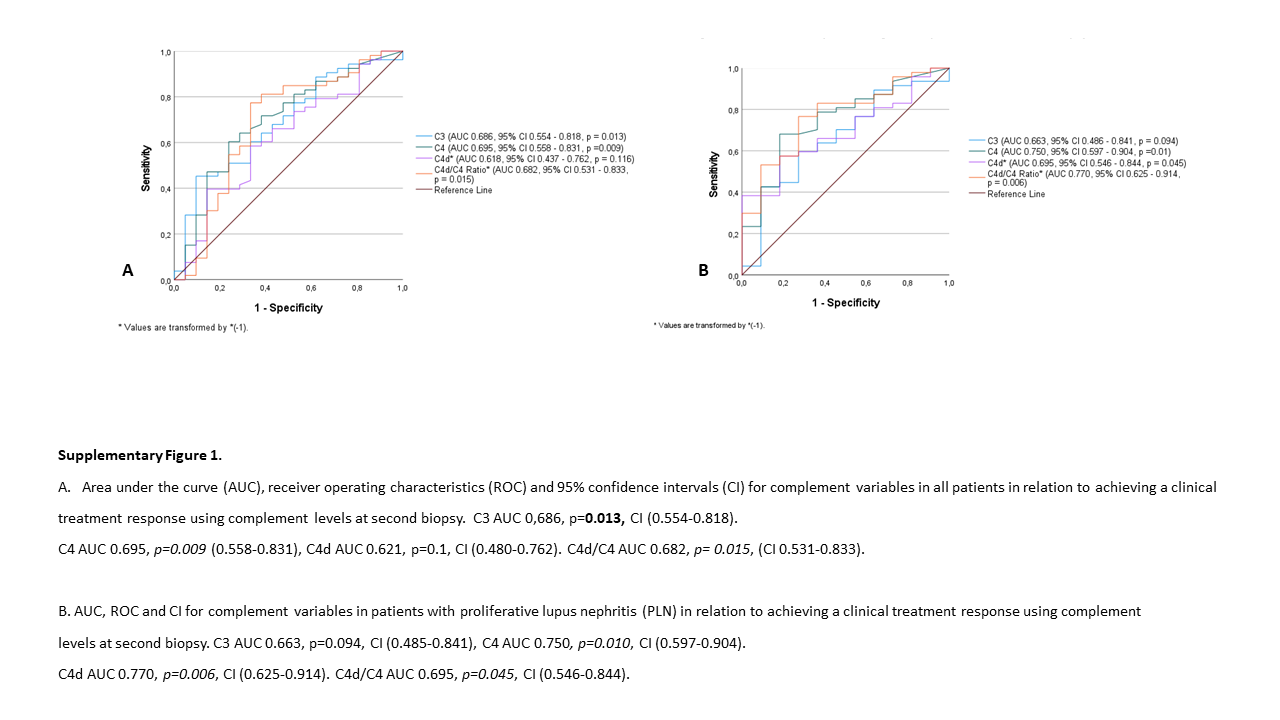
**

**Supplementary Figure S2.** Complement C4 levels at repeated biopsy in association with total numbers of C4 copy numbers and response to treatment.


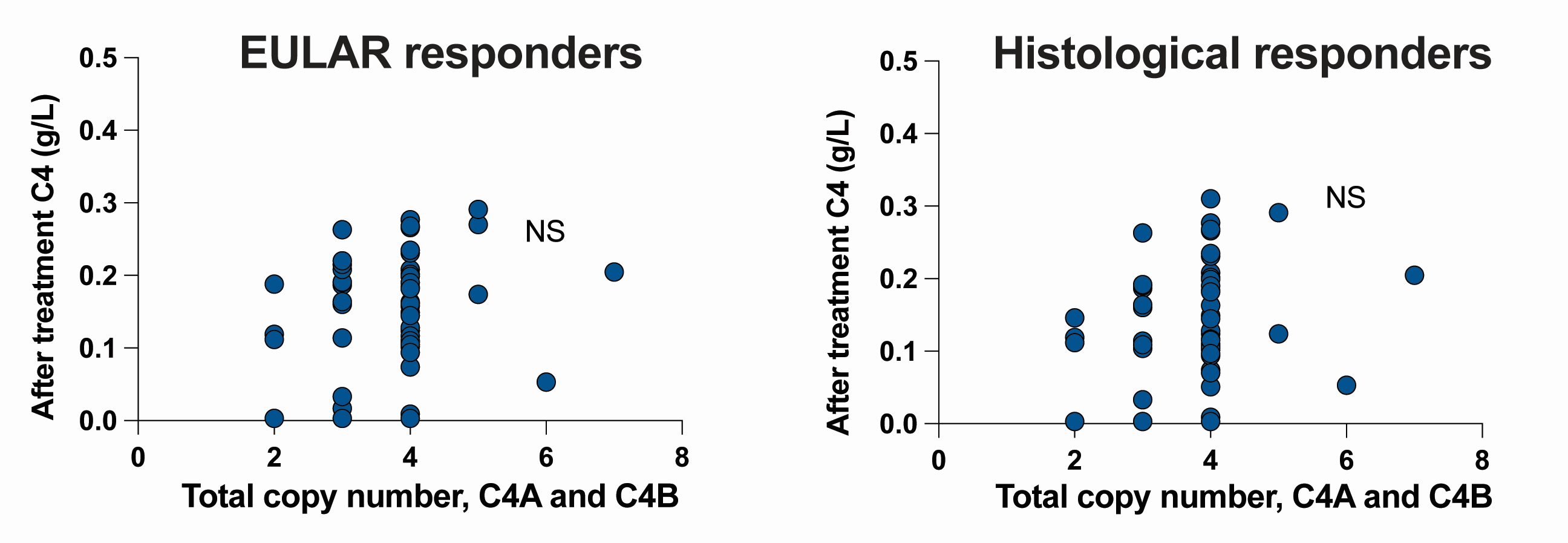

Supplement: keaf160_Supplementary_Data [file keaf160_supplementary_data.docx]
